# SHR-8068 combined with adebrelimab and bevacizumab in the treatment of refractory advanced colorectal cancer: study protocol for a single-arm, phase Ib/II study

**DOI:** 10.3389/fimmu.2024.1450533

**Published:** 2024-10-09

**Authors:** Pei Zhang, Xiaofen Li, Xin Wang, Yu Yang, Jianfei Wang, Dan Cao

**Affiliations:** ^1^ Department of Medical Oncology, Cancer Center, West China Hospital, Sichuan University, Chengdu, China; ^2^ Division of Abdominal Tumor Multimodality Treatment, Cancer Center, West China Hospital, Sichuan University, Chengdu, China; ^3^ Jiangsu Hengrui Pharmaceuticals Co., Ltd, Shanghai, China

**Keywords:** colorectal cancer, PD-L1, CTLA-4, bevacizumab, MSS

## Abstract

**Background:**

The third-line treatment for refractory colorectal cancer (CRC) has limited efficacy. This study aimed to evaluate the safety and efficacy of SHR-8068 (an anti-CTLA-4 antibody), combined with adebrelimab (an anti-PD-L1 antibody), and bevacizumab in refractory non-microsatellite instability-high (MSI-H) or proficient mismatch repair (pMMR) CRC.

**Method:**

This study is a prospective, open-label, single-center phase Ib/II clinical trial. Patients with pathologically confirmed pMMR/non-MSI-H metastatic colorectal adenocarcinoma who have failed ≥2 lines prior standard systemic treatments will be enrolled (n=36). The Ib phase will evaluate two dosing regimens of SHR-8068 in combination therapy (n=9 each dosage): SHR-8068 (1 mg per kilogram, every six weeks, intravenously) or SHR-8068 (4 mg per kilogram, every twelve weeks, intravenously) combined with adebrelimab (1200 mg, every three weeks, intravenously) and bevacizumab (7.5 mg per kilogram, every three weeks, intravenously). The efficacy and adverse events (AEs) of these regimens will be assessed to determine the recommended phase II dose (RP2D) of SHR-8068. Those of RP2D group from the phase Ib will be included in the phase II. The study will go to include 18 additional patients according to the one-sample log-rank test design in the phase II. The primary endpoint of the Ib phase is safety, with secondary endpoints including the objective response rate (ORR), progression-free survival (PFS), overall survival (OS), disease control rate (DCR), and quality of life (QOL). The primary endpoint for phase II was PFS, with secondary endpoints including ORR, OS, DCR, safety, and QOL. Identifying biomarkers to predict the efficacy of this regimen is the exploratory study endpoint.

**Discussion:**

This proof-of-concept study would provide safety and efficacy signals of this novel combination treatment for the MSS CRCs in the late-line setting. And it may offer new insights on the clinical application of dual immunotherapy combined with anti-angiogenic therapy in the MSS CRC.

## 1 Background

Colorectal cancer (CRC) ranks third in incidence and second in mortality among malignancies worldwide, with approximately 30-40% of patients eventually progressing to unresectable advanced stages (1). Current guidelines recommend chemotherapy with or without targeted agents as standard first- and second-line treatments for microsatellite stable (MSS) or proficient mismatch repair (pMMR) advanced CRC. For patients who fail second-line therapy, regorafenib, fruquintinib, or TAS-102 have shown positive results compared to placebo in clinical trials (2–4), but their efficacy is limited, with objective response rates (ORRs) less than 5% and a median overall survival (OS) of only 1.4-2.8 months, coupled with high adverse event rates. Thus, new treatment strategies are urgently needed to improve the prognosis of refractory CRC patients.

Recent evidence has shown that immunotherapy is highly effective in patients with advanced dMMR/MSI-H CRC. However, dMMR/MSI-H CRC accounts for only approximately 5% of advanced cases, limiting the application of immune checkpoint inhibitors. In patients with advanced-stage HCC or RCC, ICI doublet [typically, anti-PD-1 plus anti-CTLA4 antibodies (5, 6)] or triplet combinations [ICI doublet plus an antiangiogenic agent, such as ipilimumab plus nivolumab and cabozantinib (7)] tend to provide a more prolonged duration of response and a higher likelihood of a prolonged OS relative to antiangiogenic-ICI combinations. Anti-CTLA-4, and anti-PD-(L)1 antibodies act differentially on the immune cells (8). Programmed death-ligand 1 (PD-L1, B7-H1, CD274) expressed by tumors interacts with PD-1 on T cells to suppress T-cell effector function. CTLA-4 is a receptor on activated T lymphocytes that contains the natural ligands B7.1 and B7.2. These ligands bind to CTLA-4, releasing inhibitory signals to activated T lymphocytes and limiting their proliferation. Combination of anti-CTLA-4 and anti-PD-(L)1 therapy may prompt a stronger anti-tumor immune response leading tumor growth inhibition. Preclinical study has shown that dual CTLA-4 and PD-L1 blockade inhibits tumor growth in the microsatellite stable highly aggressive orthotopic mouse model of colon cancer due to anti-tumorigenic T cell responses mediated by CTLA-4 inhibition and M1 macrophage polarization predominantly induced by PD-L1 blockade (9). CCTG CO.26 trial of durvalumab (D) in combination with tremelimumab (T) did not show good responses in patients with advanced refractory colorectal cancer MSS type, while OS was prolonged by 2.5 months in the D+T group relative to the best-support group (10).

MSS tumors typically experience a hypoxic state, which would upregulate VEGF gene transcription and mRNA stability by stabilizing and activating hypoxia-inducible factors (11). Abnormal vasculature and elevated vascular endothelial growth factor levels lead to immunosuppression, inhibit cytotoxic T-lymphocyte (CTL) function and antigen presentation, and promote aggregation of immunosuppressive cells (12–14). Combining antiangiogenic agents with immune checkpoint inhibitors (ICIs) can potentially normalize the TME, enhancing T-cell infiltration and activity. A study combining nivolumab, plus the CTLA-4 inhibitor ipilimumab, and regorafenib, in refractory MSS CRC showed promising results in chemotherapy-refractory MSS CRC patients, with an ORR of 27.6%, a DCR of 68.2%, and a median PFS and OS of 4 and 20 months, respectively, in 29 patients (15).

SHR-8068 is an anti-CTLA-4 antibody in the clinical research phase. Adebrelimab is a high-affinity, humanized IgG4 monoclonal antibody against PD-L1.Bevacizumab (Bev) is the first approved anti-angiogenic drug and has shown synergistic effects with immune checkpoint inhibitors in the treatment of melanoma and metastatic renal cell carcinoma in previous preclinical studies (16, 17). Herein, we plan to conduct a prospective study to evaluate the safety and efficacy of SHR-8068 combined with adebrelimab and bevacizumab in advanced CRC patients to inform clinical practice.

In this study, the administration and dose selection of SHR-8068, adebrelimab, and bevacizumab were based on the previous clinical trial and authoritative guidelines. The doses for the combination of SHR-8068 (1 mg/kg every six weeks or 4 mg/kg every twelve weeks) and adebrelimab (1200 mg every three weeks) refer to the clinical trial in liver cancer (NCT05444088, data not yet public). The dose of bevacizumab (7.5 mg/kg every three weeks) was according to the 2024 NCCN Colorectal Cancer Treatment Guidelines, which endorse this regimen as safe, controlled, and effective in colorectal cancer treatment (18).

This proof-of-concept study aims to provide signals and generate preliminary evidence whether the dual immunotherapy with antiangiogenic therapy (SHR-8068 combined with adebrelimab and bevacizumab) is efficacious and well-tolerated for patients with MSS mCRC in the late-line setting.

## 2 Methods and analysis

**Figure 1:**
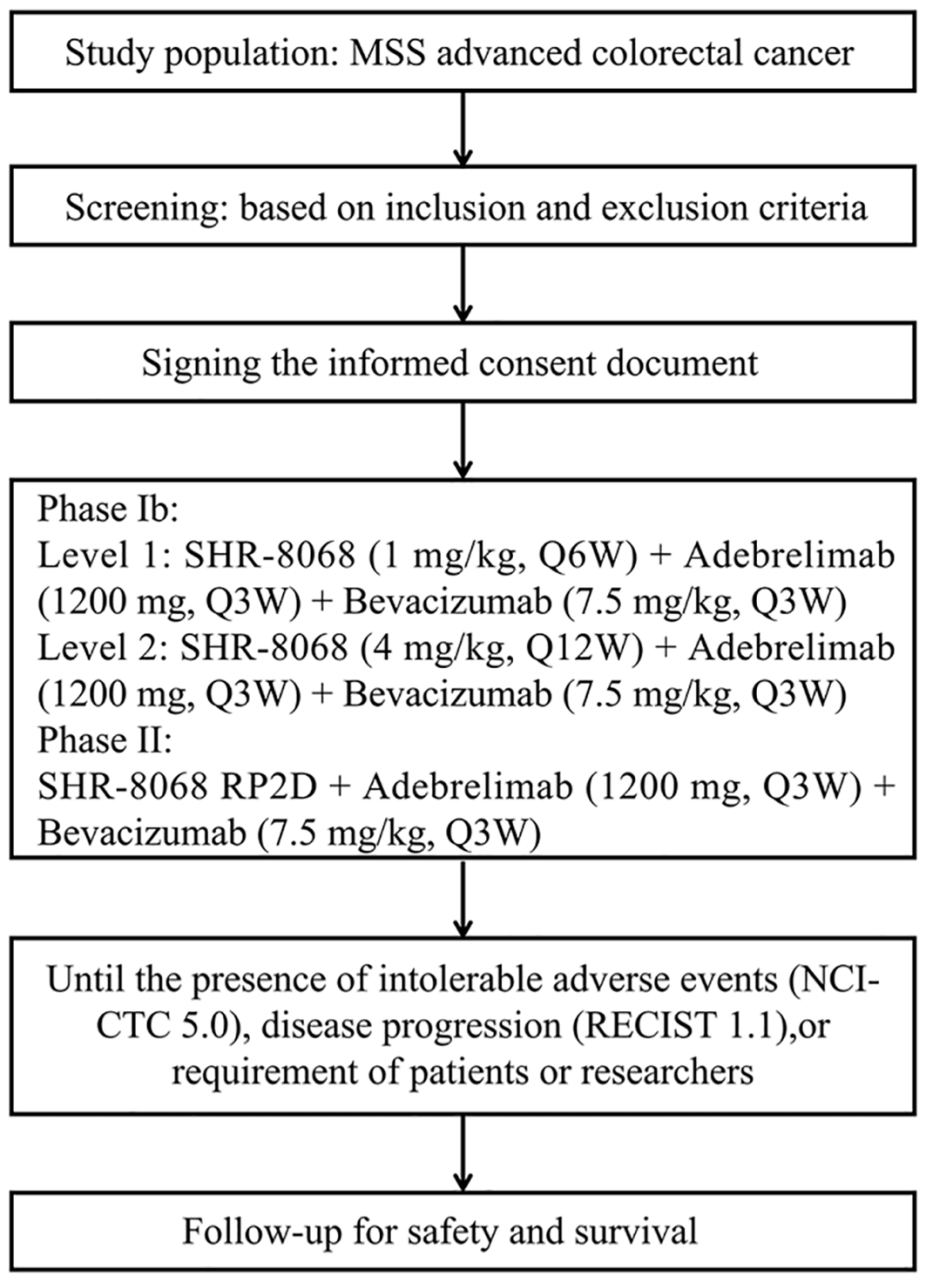
The SPIRIT flow diagram of this trial. MSS, microsatellite stable; RECIST, Response Evaluation Criteria in Solid Tumors; NCI-CTC, National Cancer Institute Common Toxicity Criteria.

This study was approved by the Biomedical Ethics Committee of West China Hospital, Sichuan University. The trial is registered on the National Clinical Trial Registry (NCT06373133). The study will be conducted in West China Hospital, Sichuan University. All participants will provide written informed consent. The trial protocol and this manuscript were developed in accordance with the Standard Protocol Items: Recommendations for Interventional Trials (SPIRIT) guidelines (**Figure 1**).

### 2.1 Study objectives

This study is a prospective, open-label, single-center, Ib/II phase clinical trial aimed at evaluating the safety and efficacy of the anti-CTLA-4 antibody SHR-8068 combined with the anti-PD-L1 antibodies adebrelimab and bevacizumab in patients with advanced refractory CRC.

This study consisted of two phases. Phase Ib focused on assessing the tolerability and safety of SHR-8068 combined with adebrelimab and bevacizumab in patients with advanced refractory CRC. Phase II evaluated the efficacy and safety of combination therapy in patients with advanced refractory CRC.

**Phase Ib**
  • Primary endpoints:
  Incidence of DLTs, TRAEs, and various grades of AEs (based on NCI-CTCAE v5.0)
• Secondary endpoints:
  ORR based on RECIST 1.1, PFS, OS, DCR, and QOL scores

**Phase II**
  • Primary endpoint:
  PFS
• Secondary endpoints:
  ORR based on RECIST 1.1, OS, DCR, safety, and QOL scores

### 2.2 Study design

**Figure 2:**
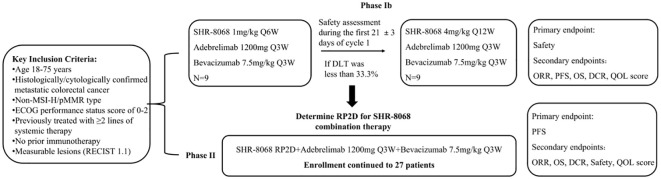
A technical roadmap for clinical trials. MSI-H, microsatellite instability-high; pMMR, proficient mismatch repair; ECOG, Eastern Cooperative Oncology Group; DLT, dose-limiting toxicity; RP2D, recommended phase 2 dose; ORR, objective response rate; PFS, progression-free survival; OS, overall survival; DCR, disease control rate; QOL, quality of life.

This study includes two phases (**Figure 2**). The phase Ib of the study aims to determine the safety of the triple-combination therapy and identify the RP2D of SHR-8068. Initially, 9 participants will receive adebrelimab (1200 mg, every three weeks, intravenously) plus SHR-8068 (1 mg/kg, every six weeks, intravenously) plus bevacizumab (7.5 mg/kg, every three weeks, intravenously). The tolerability observation period was 21 ± 3 days. If the regimen is intolerable (dose-limiting toxicity (DLT) incidence ≥ 33.3%), the researchers will assess whether to adjust the dosage, or frequency of administration, or terminate the study. If tolerable (DLT incidence less than 33.3%), another nine participants will receive adebrelimab (1200 mg, every three weeks, intravenously) plus SHR-8068 (4 mg per kilogram, every twelve weeks, intravenously) plus bevacizumab (7.5 mg per kilogram, every three weeks, intravenously). After completing the tolerability observation (21 ± 3 days), if this dose is intolerable (DLT incidence ≥ 33.3%), the RP2D of SHR-8068 will be determined to be 1 mg/kg, Q6W. If tolerable (DLT incidence < 33.3%), the efficacy and adverse event (AE) profiles of both dose groups will be comprehensively evaluated to determine the RP2D of SHR-8068 for continuation into phase II.

The phase II will assess the efficacy of the combination with RP2D on 27 patients (details in 2.5 sample size), including those of RP2D group from the phase Ib.

The treatment will continue until disease progression (PD), intolerable adverse events (AEs), as required by patients and researchers, or up to 24 months.

### 2.3 Eligibility and enrollment

Patients diagnosed with non-MSI-H/pMMR metastatic colorectal cancer, and failure of at least two lines of standard systemic therapy will be enrolled in this study. Further key inclusion criteria are 1) 18 to 75 years old; 2) an ECOG performance status of 0-2; 3) a predicted survival of at least 12 weeks; 4) at least one measurable tumor lesion. Major exclusion criteria include 1) a history of other malignancies within the past five years; 2) known CNS metastases; 3) severe cardiovascular conditions; 4) the presence of uncontrolled complications; 5) significant autoimmune diseases or immunodeficiency syndrome. Details of inclusion and exclusion criteria are listed in **Table 1** and **Table 2**.

**Table 1:** Details of the inclusion criteria for this study.

Inclusion Criteria
1. Pathologically confirmed metastatic CRC;
2. Age 18-75 years, both sexes;
3. Predicted survival time ≥12 weeks;
4. Eastern Cooperative Oncology Group performance status (ECOG) score 0-2;
5. Failed second-line or higher standard-of-care systemic therapy and treated with oxaliplatin, irinotecan, and fluorouracil if included in first-line therapy;
6. Liver tumor burden ≤50% (assessed by enhanced CT or MRI);
7. No serious complications (perforation, obstruction, massive bleeding, etc.) in the primary lesion;
8. At least one measurable objective tumor lesion per RECIST 1.1 (≥1 cm by spiral CT, ≥2 cm by conventional CT or MRI, within 28 days before enrollment);
9. Adequate organ function within 1 week before enrollment (no blood transfusion or medical support within 14 days): Hemoglobin ≥90 g/L, Platelet ≥75×10^9/L,3) White blood cell ≥3.0×10^9/L, Neutrophil ≥1.5×10^9/L, Total bilirubin ≤1.5×UNL, Serum creatinine ≤1.5×UNL
10. ALT, AST ≤5×UNL; Active hepatitis B or C with antiviral therapy for ≥14 days before the first dose, with controlled HBV DNA and HCV RNA levels;
11. Willing to provide written informed consent;

CRC, Colorectal Cancer; ECOG, Eastern Cooperative Oncology Group; CT, Computed Tomography; MRI, Magnetic Resonance Imaging; RECIST, Response Evaluation Criteria In Solid Tumors; UNL, Upper Normal Limit; HBV, Hepatitis B Virus; HCV, Hepatitis C Virus; DNA, Deoxyribonucleic Acid; RNA, Ribonucleic Acid.

**Table 2:** Details of the exclusion criteria for this study.

Exclusion Criteria
1. History of other malignancies with DFS <5 years (except cured basal cell carcinoma of the skin, cured carcinoma *in situ* of the cervix, and gastrointestinal cancer proven cured by endoscopic mucosal resection);
2. Participated in other drug clinical trials within four weeks;
3. Known CNS metastases or a history of CNS metastases before screening; for clinically suspected CNS metastasis, contrast-enhanced CT or MRI is required within 28 days before informed consent to rule out CNS metastasis;
4. Long history of chronic diarrhea or complete intestinal obstruction;
5. Urinalysis showing urinary protein ≥2+ or 24-hour urinary protein ≥1 g;
6. Drug-uncontrolled hypertension (systolic BP >140 mmHg or diastolic BP >90 mmHg);
7. History of severe bleeding (>30 ml per episode) within 3 months, hemoptysis (>5 ml per episode) within 1 month, or thromboembolic events (including pulmonary embolism, cerebral infarction, etc.) within 12 months;
8. Undergone surgical treatment (excluding biopsy) within 6 weeks or unhealed surgical incisions;
9. Long-term unhealed wounds or incompletely healed fractures;
10. Imaging showing the tumor has invaded vital blood vessels or the tumor has a high possibility of invading vital blood vessels during treatment, which may cause fatal bleeding;
11. History of unstable angina pectoris; newly diagnosed angina pectoris within 3 months before screening or myocardial infarction within 6 months before screening; arrhythmias (including QTcF ≥450 ms in men and ≥470 ms in women) requiring long-term use of antiarrhythmic drugs, and NYHA grade ≥II cardiac dysfunction;
12. Abnormal coagulation function and bleeding tendency (INR within the normal range without anticoagulant therapy must be met within 14 days before the first medication); patients treated with anticoagulants or vitamin K antagonists such as warfarin, heparin, or their analogs; low-dose warfarin (1 mg orally once daily) or low-dose aspirin (at a dose of up to 100 mg daily) for preventive purposes were allowed if the INR was 1.5 or less;
13. Active or prior autoimmune disease or risk (e.g., organ transplant requiring immunosuppressive therapy) that may relapse; however, participants with type 1 diabetes, hypothyroidism receiving hormone-replacement therapy only, or skin conditions not requiring systemic treatment (e.g., vitiligo, psoriasis, or alopecia) were allowed;
14. History of interstitial lung disease or noninfectious pneumonia, such as symptomatic disease or a previous pulmonary history that may preclude assessment or management of study-drug-related pulmonary toxicity;
15. History of active pulmonary tuberculosis infection within 1 year before the first dose of the study drug; a history of active pulmonary tuberculosis infection more than 1 year before was considered eligible if they had no evidence of current active pulmonary tuberculosis, as assessed by the investigator;
16. Severe uncontrolled medical illness or acute infection (fever >38°C);
17. Required systemic treatment with corticosteroids (>10 mg/day prednisone equivalent dose) or other immunosuppressive drugs within 14 days prior to administration of the study drug. Note: In the absence of active autoimmune disease, inhaled or topical steroid hormones, or adrenal hormone replacement therapy with an equivalent dose of ≤10 mg prednisone per day, were allowed. Short-term (≤7 days) use of glucocorticoids was permitted for preventive treatment (e.g., contrast allergy) or for the treatment of nonautoimmune conditions (e.g., delayed hypersensitivity from a contact allergen);
18. Treated with any antibody/drug (e.g., anti-PD-1, anti-PD-L1, anti-CTLA-4, anti-OX-40, anti-CD137, anti-TIM-3, anti-LAG-3 antibodies, etc.) targeting a T-cell coregulatory protein (immune checkpoint);
19. History of allergic or hypersensitivity reactions to study drug components;
20. Immunodeficiency diseases or HIV infection;
21. Pregnant or lactating women or patients of childbearing potential (men or women who have been without menstruation for less than 1 year) who are unwilling to use contraception;
22. Concomitant diseases that, according to the investigator’s judgment, seriously endanger the patient’s safety or prevent the patient from completing the study;
23. Deemed by the investigator to be ineligible for participation in the trial;

DFS, Disease-Free Survival; CNS, Central Nervous System; CT, Computed Tomography; MRI, Magnetic Resonance Imaging; BP, Blood Pressure; QTcF, QT Interval Corrected for Heart Rate by Fridericia’s Formula; NYHA, New York Heart Association; INR, International Normalized Ratio; HIV, Human Immunodeficiency Virus; PD-1, Programmed Cell Death Protein 1; PD-L1, Programmed Death-Ligand 1; CTLA-4, Cytotoxic T-Lymphocyte-Associated Protein 4; OX-40, Tumor Necrosis Factor Receptor Superfamily Member 4; CD137, Cluster of Differentiation 137; TIM-3, T-Cell Immunoglobulin and Mucin-Domain Containing-3; LAG-3, Lymphocyte-Activation Gene 3.

Information collection and medical examination including demographic data, tumor history, medical history, quality of life scores, tumor imaging, echocardiography, HIV/HBV/HCV tests, drug allergy history, ECOG score, physical examination, vital signs, ECG, complete blood count, urinalysis, stool analysis, blood biochemistry, coagulation function, cardiac enzyme profile, pregnancy test for female patients, and thyroid function test are required to undergo during the screening period (**Table 3**).

**Table 3:** Schedule of patient visits and assessments.

Study Step/Items of follow-up	Screening Period		Treatment Period (42/84 days per cycle)					Post-treatment		
			First Cycle (C1)			Second Cycle and Subsequent Cycles (C2-Cx)		Post-treatment/Exit		
	Within 4 weeks pre-dosing	Within 1 week pre-dosing	D1	D8 (± 3 days)	D22/(D43/D64) (± 3 days)	D1 (± 3 days)	D22/(D43/D64) (± 3 days)	End of treatment/Exit (14/+3 days)	Safety Follow-up (30 days/visit)	Survival Follow-up (3 months/visit)
Laboratory Examination										
Blood Chemistry		√		√	√	√	√	√	√	
Coagulation Test		√			√	√	√	√	√	
Tumor Markers		√	Each imaging examination						√	
Myocardial Enzyme Spectrum Test		√			√			√		
HIV, Hepatitis B, Hepatitis C Tests	√		If the subject is a Hepatitis B virus carrier, monitor the viral load every cycle and continue antiviral treatment					√		
Pregnancy Test		√							√	
Thyroid Function Test		√			√			√	√	
Pituitary-Adrenal Axis Test (Optional)		√						√		
Efficacy Evaluation										
Tumor Imaging Examination	√		Once every 6 weeks (± 7 days). After 8 months, adjust to once every 12 weeks (± 7 days)					√		
Adverse Events/ Concomitant Medications										
Adverse Events	From signing the informed consent form until at least 30 days after the last dose									
Concomitant Medications	From signing the informed consent form until at least 30 days after the last dose									
Follow-up of Survival										
Disease Progression								Once every 3 months (± 7 days) imaging evaluation until disease progression or initiation of other tumor treatments (non-imaging PD patients)		
Survival Status										√
Samples Collection and Testing										
Blood Sample Collection and Testing		√								
Tumor Sample Collection and Testing		√								

C1:, Cycle 1; C2-Cx, Cycle 2 and subsequent cycles; HIV, Human Immunodeficiency Virus; CT, Computed Tomography; MRI, Magnetic Resonance Imaging; PD, Progressive Disease; HBV, Hepatitis B Virus; HCV, Hepatitis C Virus.

Patients are included after giving written informed consent and finally enrolled in the clinical trial when the inclusion and exclusion criteria are met. Qualified study personnel will provide patients with comprehensive information regarding the study’s purpose, procedures, potential risks, benefits, and the rights of patients. The informed consent process will comply with IRB guidelines and local regulations to protect patient rights and privacy.

### 2.4 Analysis and evaluation

#### 2.4.1 MSI/MMR analysis criteria

Tumor MSI/MMR was assessed per polymerase chain reaction (PCR) and immunohistochemistry (IHC), respectively, prior to screening.

According to the recommendations of National Cancer Institute (19), MSI was identified by the panel of five microsatellites markers: BAT-25, BAT-26, D2S123, D5S346 and D17S250. MSI-H is reported if two or more markers show instability (i.e., have insertion/deletion mutations), MSI-L is reported if only one marker shows instability, and MSS indicates that no markers were positive.

IHC for four MMR proteins (MLH1, PMS2, MSH2 and MSH6) was performed to identify MMR status. pMMR (proficient MMR) was defined as all four MMR proteins present. And if one or more of four proteins are absent, it is classified as dMMR (deficient MMR). The processed IHC slides were evaluated by two pathologists.

The microsatellites status assessed with PCR-based testing and MMR status evaluated by IHC are generally highly consistent (20).

#### 2.4.2 Efficacy and toxicity assessment procedures

Evaluations of tumor response to treatment utilize images from MRI or contrast-enhanced (preferred) CT will be performed every 6 weeks (adjusted to every 12 weeks after 8 months) according to the Response Evaluation Criteria in Solid Tumors (RECIST) version 1.1 until disease progression or intolerable toxicity. The first tumor evaluation should be conducted at least 6 weeks after the first dose. For patients showing initial radiological PD requires confirmation with repeat imaging after ≥4 weeks if patients are clinically stable to account for the possibility of pseudo-progression.

Regarding to the safety assessments, treatment toxicity will be evaluated at each study visit and AEs will be reported according to the Common Terminology Criteria for Adverse Events (CTCAE) version 5.0. In the first medication cycle, ECOG score, physical examination, vital signs, ECG, complete blood count, urinalysis, blood biochemistry will be taken on D8, D22(for 6-week regimen), D43, and D64 (for 12-week regimen). From the second cycle onward, routine examinations (vital signs, ECG, complete blood count, urinalysis, stool analysis, blood biochemistry, coagulation function) will be conducted on D1, D22(for 6-week regimen), D43, and D64 (for 12-week regimen). Upon treatment termination, a comprehensive assessment will be performed, and the reasons for discontinuation will be recorded. Serious AEs (SAEs) occurring during treatment or until 90 days after the last dose of treatment should be recorded and reported immediately and not exceeding 24h after the knowledge of SAEs.

For patients with disease progression, survival status should be assessed every 3 months after treatment discontinuation. Survival data will be calculated.

A more detailed study schedule is shown in **Table 3**.

#### 2.4.3 Biomarker exploration

Patients are asked to consent to allow access to any available archival tumor tissue previously obtained. And blood samples will be collected at baseline (pre-treatment). Tumor tissue and blood sample will undergo next-generation sequencing (NGS) to explore potential predictive markers for response to the therapy.

### 2.5 Sample size

This study consists of two phases. In phase Ib, two dose groups will enroll 9 participants each to determine the RP2D of SHR-8068 in combination therapy. Participants in the RP2D dose group from phase Ib will continue into phase II.

Phase II employs a one-sample log-rank test design. Based on the results of three phase III trials of third-line standard treatments for colorectal cancer (CORRECT, FRESCO, and TERRA studies), the null hypothesis is the median PFS of 2 months. And the expected median PFS is assumed to be 4 months. With M0 = 2, M1 = 4, a power of 0.9, and a one-sided α of 0.05, the sample size is estimated using the one-sample log-rank test in PASS. With an enrollment period of 12 months and a follow-up period of 12 months, a total of 24 participants were needed. Considering a 10% dropout rate, the total number of participants enrolled should be 27.

The study will last a total of 2.5 years, with participant enrollment occurring in the first year.

### 2.6 Dose modifications

During the study, dose adjustments for adebrelimab and SHR-8068 were not allowed, and only interruptions were permitted, with a maximum of 12 weeks (**Table 4**). If a serious adverse event or grade 3/4 nonserious adverse event related to bevacizumab occurs, it should be suspended. Bevacizumab can be resumed if the event resolves to grade 1 and is deemed beneficial to the patient. If toxicity delays treatment for more than 6 weeks, bevacizumab should be discontinued (**Table 5**).

**Table 4:** Recommended action for SHR-8068 and/or adebrelimab for adverse events.

Immune-Related Adverse Event	Severity	Treatment Adjustment
Colitis	Grade 2	Suspend use
	Grade 3 or 4	Permanently discontinue
Hepatitis without liver metastasis or with liver metastasis (non-HCC)	AST or ALT elevated to 3-5x ULN, or bilirubin elevated to 1.5-3x ULN	Suspend use
	AST or ALT elevated >5x ULN, or bilirubin elevated >3x ULN	Permanently discontinue
Hepatitis associated with HCC	Baseline AST/ALT 1-3x ULN, elevated to 5-10x ULN	Suspend use
	AST/ALT elevated >10x ULN, or bilirubin elevated >3x ULN	Permanently discontinue
Exfoliative dermatitis	Suspected SJS, TEN, or DRESS	Suspend use
	Confirmed SJS, TEN, or DRESS	Permanently discontinue
Endocrine diseasec	Grade 3 or 4	Suspend use until clinically stable, or permanently discontinue if severe
Pneumonitis	Grade 2	Suspend use
	Grade 3 or 4	Permanently discontinue
Nephritis with renal dysfunction	Grade 2 or 3 elevated creatinine	Suspend use
	Grade 4 elevated creatinine	Permanently discontinue
Neurotoxicity	Grade 2	Suspend use
	Grade 3 or 4	Permanently discontinue
Myocarditis	Grade 2, 3, or 4	Permanently discontinue
Ocular toxicity	≥Grade 2 toxicity not resolved to Grade 1 with topical treatment in 2 weeks, or requiring systemic treatment	Permanently discontinue
Infusion-related reaction	Grade 1 or 2	Interrupt infusion or reduce infusion rate
	Grade 3 or 4	Permanently discontinue
Other immune-related adverse reactions	Grade 2 or 3	Suspend use
	Grade 3 or 4 encephalitis	Permanently discontinue
	Grade 3 or 4 Guillain−Barre syndrome	Permanently discontinue
	Grade 4 or recurrent Grade 3	Permanently discontinue

ALT, Alanine aminotransferase; AST, Aspartate Aminotransferase; DRESS, Drug Rash with Eosinophilia and Systemic Symptoms; SJS, Stevens-Johnson Syndrome; TEN, Toxic Epidermal Necrolysis; ULN, Upper Limit of Normal.

**Table 5:** Recommended action for bevacizumab for adverse events.

Bevacizumab Related Toxicity	Grade	Management Measures
Hypertension	Grade 1	Do not suspend
	Grade 2	Suspend bevacizumab, initiate antihypertensive therapy, resume bevacizumab when BP <140/90 mmHg
	Grade 3	Suspend bevacizumab, initiate antihypertensive therapy, resume bevacizumab when BP <140/90 mmHg; discontinue bevacizumab if uncontrolled
	Grade 4 (including hypertensive encephalopathy)	Discontinue bevacizumab
Bleeding	Grade 1/2 nonpulmonary and non-CNS	Do not suspend
	Grade 3 nonpulmonary and non-CNS	Suspend bevacizumab until all criteria met: 1) bleeding stopped and hemoglobin stable; 2) no risk factors for bleeding; 3) no anatomical or pathological conditions increasing bleeding risk
	Grade 4 nonpulmonary and non-CNS	Discontinue bevacizumab
	Grade 1 pulmonary or CNS	Suspend bevacizumab until all criteria met: 1) bleeding symptoms resolved and hemoglobin stable; 2) no risk factors for bleeding; 3) no anatomical or pathological conditions increasing bleeding risk
	Grade 2 or higher pulmonary or CNS	Discontinue bevacizumab
	Repeated Grade 3	Discontinue bevacizumab
Venous Thromboembolism	Grade 1/2	Do not suspend
	Grade 3 or asymptomatic Grade 4	If planned anticoagulant therapy duration ≤2 weeks, do not suspend; if >2 weeks, resume bevacizumab after 2 weeks if all conditions met: 1) INR within normal range; 2) stable dose of low molecular weight heparin, warfarin, or other anticoagulant; 3) no Grade 3/4 bleeding events
	Grade 4 with symptoms	Discontinue bevacizumab
	Any grade	Discontinue bevacizumab
Arterial Thromboembolism	Any grade	Discontinue bevacizumab
Congestive Heart Failure	Grade 1/2	Do not suspend
	Grade 3	Suspend bevacizumab until Grade ≤1
	Grade 4	Discontinue bevacizumab
Proteinuria	Grade 1	Continue bevacizumab and perform 24-hour urine protein quantification before next dose if proteinuria 2+; perform 24-hour urine protein quantification before next dose if proteinuria 3+
	Grade 2	Suspend bevacizumab if proteinuria ≥2 g/24 h; resume bevacizumab if proteinuria <2 g/24 h
	Grade 3	Suspend bevacizumab if proteinuria ≥2 g/24 h; resume bevacizumab if proteinuria <2 g/24 h
	Grade 4	Discontinue bevacizumab
Gastrointestinal Perforation	Any grade	Discontinue bevacizumab
Fistula	Tracheoesophageal fistula of any grade	Discontinue bevacizumab
	Grade 4 (except tracheoesophageal)	Discontinue bevacizumab
Intestinal Obstruction	Grade 1 without medical intervention	Continue bevacizumab if Grade 1 without medical intervention
	Grade 2 or higher	Discontinue bevacizumab
Wound Dehiscence	Any grade (requiring medical or surgical treatment)	Discontinue bevacizumab
Reversible Posterior Leukoencephalopathy Syndrome	Any grade (confirmed by MRI)	Discontinue bevacizumab

BP, Blood Pressure; CNS, Central Nervous System; INR, International Normalized Ratio; AST, Aspartate Aminotransferase; ALT, Alanine Aminotransferase; ULN, Upper Limit of Normal; SJS, Stevens-Johnson Syndrome; TEN, Toxic Epidermal Necrolysis; DRESS, Drug Reaction with Eosinophilia and Systemic Symptoms; HCC, Hepatocellular Carcinoma; MRI, Magnetic Resonance Imaging.

After a total of 35 doses of adebrelimab, and/or after 18 doses of SR-8068 (1mg/kg, Q6W)/9 doses of SHR-8068(4mg/kg, Q12W), treatment should be terminated. If SHR-8068 is terminated prematurely due to toxic intolerance, adebrelimab alone or in combination with bevacizumab may be considered. However, if adebrelimab is discontinued due to toxic intolerance or other reasons, SHR-8068 and bevacizumab therapy should be terminated.

### 2.7 DLT determination

During the tolerability observation period (21 ± 3 days), any event judged by the investigator to be related to the study drug (grading criteria based on NCI-CTCAE 5.0) was considered as a DLT if it met any of the following criteria:

1. Grade 4 hematologic toxicity (excluding grade 4 lymphocyte count reduction unless accompanied by opportunistic infection).
2. Grade ≥3 thrombocytopenia with bleeding.
3. Grade ≥3 neutropenia with fever (defined as absolute neutrophil count [ANC]<1000/mm³ with a single temperature of 38.3°C or a sustained temperature of 38°C for more than 1 hour).
4. Grade ≥3 nonhematologic toxicity (excluding abnormal laboratory test results), with the following exceptions: fatigue/asthenia improving to≤grade 2 within 3 days; untreated grade ≥3 diarrhea, nausea, or vomiting; untreated grade ≥3 rash not managed with corticosteroids or anti-inflammatory drugs.
5. Grade ≥3 nonhematologic laboratory abnormalities requiring medical intervention, leading to hospitalization, or persisting for ≥7 days.
6. Drug-related toxicity causing a delay in the administration of adebrelimab or SHR-8068 for >7 days.
7. Drug-related toxicity leading to the discontinuation of treatment during the DLT observation period.

### 2.8 Statistics

This study will follow the ITT principle, analyzing all participants who signed informed consent. The analysis populations included the FAS, PPS, and SAS. Efficacy analyses will be performed in the FAS and PPS populations, while safety analyses will be performed in SAS.

Baseline characteristics will be described using the mean ± SD for continuous variables and frequency (percentage) for categorical variables. Survival data will be analyzed using the Kaplan−Meier method to estimate PFS and OS curves, with medians and 95% CIs calculated. The ORR and DCR will be descriptively analyzed, with 95% CIs calculated using the Clopper–Pearson method. The incidence of AEs, treatment-related AEs, SAEs, and AEs leading to study withdrawal will be summarized.

*Post-hoc* analysis of objective response, PFS, and OS across different groups will be performed. Univariate and Cox multivariate analyses will be used to assess the impact of baseline characteristics on PFS and OS.

All the statistical analyses will be conducted using SPSS version 22 or above.

## 3 Discussion

Considering that the current therapies and trialed regiments for patient with mCRC obtained unsatisfactory survival outcomes (12, 21, 22), seeking further effective treatment strategy to extend life is the urgent requirement. Herein, we have a study design to evaluate the safety and efficacy of the dual-immunology therapy (CTLA-4 inhibitor, SHR-8068 and anti-PD-L1 antibody, adebrelimab) combined with antiangiogenic agent (bevacizumab) in late-line line for advanced CRC patients.

Previously, the REGONIVO study conducted by Japanese research team has demonstrated the potential of combining anti-angiogenic drug(regorafenib) with PD-1 inhibitor (nivolumab) for the treatment of MSS CRC, which achieved an ORR of 36%, and a median PFS of 7.9 months (12). However, efficacy of this combination in the North American population did not reproduce findings in the Japanese population, obtaining an ORR of 7.1% (23). It is consistent with other similar combination regiment and some real-world studies showing disappointing results (13, 14, 24). These findings put a mist over the treatment of immunotherapy with anti-angiogenic agent for MSS CRCs. The RIN trial took the first step to adopt a combination of dual-immunotherapy (nivolumab plus ipilimumab) with regorafenib in refractory CRCs (15). And it shows promising results with an ORR of 27.6% and a median PFS of 4 months in the total population. However, like previous similar studies mentioned above, the regiment brings limited benefit to patients with liver metastasis. Regrettably, due to the data of the baseline liver tumor burden not released, whether the combination of immunotherapy with anti-angiogenic therapy is only effective for CRCs without liver metastasis is unclear. In the current study, we limit the liver tumor burden in the enrolled patients to less than 50% (assessed by enhanced CT or MRI). This specific inclusion criterion is in the consideration of avoiding enrolling patients with large hepatic tumor loads, who are highly unlikely to derive clinical benefit from the study treatment based on data from previous studies, and to further identify whether this dual-immunotherapy plus anti-angiogenic agent could bring clinical benefit in the CRC patients with low hepatic tumor loads.

Besides the efficacy, toxicity is the most important factor to consider for the third- or later-line therapy choice. Immune-related adverse events typically involve the skin, gastrointestinal, hepatic, and endocrine systems (25). A meta-analysis has reported that the overall AE rate of monotherapy with PD-1 antibody in pMMR CRCs were 54% (26). While all patients enrolled in the CCTG CO.26 trial occurred therapeutic toxicity after receiving durvalumab with tremelimumab, and 64% of patients experienced ≥ Grade 3 AEs. The most common toxicities above 3 degrees were fatigue and abdominal pain (10). Increases in CTLA-4 inhibitor dose/exposure may lead to higher incidence of Grade 3/4 TRAEs, hepatic irAEs, and gastrointestinal irAEs (27). As for the anti-angiogenic agent, common adverse events associated with bevacizumab includes hypertension, thromboembolism, hemorrhage, and proteinuria (28). A meta-analysis shows that the relative risks for bevacizumab were relatively low in comparison to regorafenib, in the case of diarrhea and febrile neutropenia (29). While it should be noticed that bevacizumab may increase the risk of upper gastrointestinal bleeding and colitis when combined with more than one ICI (30). Individuals who are more vulnerable to the risks of the treatment should be excluded per the protocol. Meanwhile, a comprehensive safety monitoring would be conducted in this study to ensure the safety of patients and receive timely treatment.

In order to ensure the safety and maximize the benefits of the combinations for patients, we implemented a safety lead-in phase for two doses. We will take both safety and efficacy data into account to select the RP2D for Phase II expansion, rather than exploring only a fixed dose. Moreover, to identify the survival benefit signal of the treatment, the phase II employs a one-sample log-rank test design based on the primary endpoint of PFS. It was used to determine whether the novel combination of SHR-8068 plus adebrelimab and bevacizumab should be considered for further testing. The one-sample log-rank test is the method of choice for single-arm Phase II trials with time-to-event endpoint. It allows to compare the survival of the patients to a reference survival curve that typically represents the expected survival under standard of care (31). Further larger randomized clinical trial would be needed (dual-immunotherapy + Bev vs. PD-L1 antibody + Bev vs. BSC) in the future if the primary endpoint of this study was met.

This study would offer more compelling evidence on the clinical application of dual immunotherapy combined with anti-angiogenic therapy in the MSS CRC with or without liver metastasis. Additionally, biomarker exploration in the study would give deeper understanding of the dual-immunotherapy combined with anti-angiogenic drug in CRCs, and provide better insights in predictive markers for response to the therapy.
